# Cofilin-1 induces acute kidney injury via the promotion of endoplasmic reticulum stress-mediated ferroptosis

**DOI:** 10.1007/s13577-023-00949-9

**Published:** 2023-08-07

**Authors:** Sihao Lin, Jie Wang, Bin Cao, Yang Huang, Xujun Sheng, Yingjian Zhu

**Affiliations:** 1 Department of Urology, Jiading Branch of Shanghai General Hospital, Shanghai Jiao Tong University School of Medicine, 800 Huangjiahuayuan Road, Shanghai, 201803 People’s Republic of China; 2 Department of Urology, Chengmai County People Hospital, Hainan, 571900 People’s Republic of China; 3 Department of Urology, Xinhua Hospital Affiliated to Shanghai Jiao Tong University School of Medicine, Shanghai, 200092 People’s Republic of China

**Keywords:** Cofilin-1, Acute kidney injury, Endoplasmic reticulum, Ferroptosis

## Abstract

Ischemia–reperfusion injury (IRI) leads to acute kidney injury (AKI), which poses serious threat to public health and society. Many clinical studies were conducted to evaluate several biomarkers in AKI, among which Cofilin-1 remains to be a very promising one. To explore the potential mechanism of Cofilin-1 in AKI, we established an oxygen-glucose-deprivation (OGD)-induced AKI cell model. The overexpression and knock-down Cofilin-1 were used for gain- and loss-of-function. Pharmacological inhibitors were employed to study the related pathways. The results showed that Cofilin-1 was significantly upregulated in AKI cells, knocking down Cofilin-1 protected cells against the effect of OGD treatment and alleviated AKI phenotypes. Overexpression of Cofilin-1 might induce AKI by triggering ferroptosis, inhibiting NF-κB signaling or ER stress pathway attenuated Cofilin-1 induced lipid peroxidation and AKI. We also validated our findings in IRI-induced AKI mouse models in vivo. Our work elucidated that Cofilin-1 might induce AKI via promoting ER stress-mediated ferroptosis and argues it as a biomarker for early diagnosis of AKI. We also expect to offer novel insights on future therapeutic interventions.

**Supplementary Information:**

The online version contains supplementary material available at 10.1007/s13577-023-00949-9.

## Introduction

Acute kidney injury (AKI) is a complex renal disorder, while multifactorial, primarily attributed to ischemia–reperfusion injury (IRI) [1]. AKI occurs in 10–15% of hospitalized patients and over 50% patients are under intensive care [2], which poses serious threats to public health and society. Understanding how IRI leads to AKI pathogenesis and the detailed molecular mechanisms will offer novel insights into developing effective clinical intervention strategies. Renal IRI involves multifold alterations in tissue morphology, elevated cellular reactive oxygen species (ROS) and lipid peroxidation, and cellular metabolism dysfunction [1, 3].

Although most studies have focused on how oxidative stress triggers cellular apoptosis and autophagy, new mechanism continue to emerge. In 2012, Dixon et al. discovered ferroptosis as an iron-dependent type of programmed cell death [4]. Ferroptosis is triggered by elevated lipid peroxidation when ROS accumulate in the cellular niche [5]. Small molecules, such as erastin are known to stimulate ferroptosis where others like ferrostatin-1 (Fer-1) inhibit it [4, 6]. Interestingly, ferroptosis has recently been proposed to play a vital role in IRI-induced AKI, making it a promising target for clinical intervention [7]. In addition, [8–10] [11, 12]it has become increasingly accepted that endoplasmic reticulum (ER) homeostasis and the unfolded protein response (UPR) are evident in AKI samples [13]. However, the detailed mechanisms by which ER stress contributes to AKI pathogenesis and whether ferroptosis is involved remain elusive.

The canonical NF-κB family of transcription factors are known to regulate many biological events including inflammation. Therefore, it is almost intuitive to find activation of NF-κB signaling in cultured renal cells and in endogenous glomerular cells upon renal injury or exposure to inflammatory stimuli [14, 15]. Interestingly, NF-κB was recently proposed to modulate ER stress and ferroptosis [16, 17]. However, little is known about whether NF-κB regulates ER stress and ferroptosis in AKI.

More recently, many clinical studies were conducted to evaluate several biomarkers in the early diagnosis of AKI, among which Cofilin-1 remains to be a very promising one [18]. Cofilin-1 was originally identified as an actin-regulating protein and was essential for development because cofilin-1-null mice were embryonically lethal [18, 19]. Importantly, Cofilin-1 was evidently upregulated in cultured human kidney HK-2 cell line upon exposure to hydrogen peroxide (H_2_O_2_) and arsenic trioxide (ATO) [20]. However, the detailed mechanisms by which Cofilin-1 contributes to AKI are yet to be determined.

The current work attempts to investigate the possible role of Cofilin-1 in AKI and potential molecular mechanism using an oxygen-glucose-deprivation (OGD)-induced AKI and mouse AKI models in vivo, and elucidate how Cofilin-1 upregulation could render AKI and related cellular defects, supporting the notion that Cofilin-1 is a candidate marker and therapeutic target of AKI. Our research also expects to offer novel insights on designing therapeutic strategies in the future.

## Methods and materials

### Cell line and constructs

A stable human kidney HK-2 cell line was used in this study (Cell Bank of Shanghai Biology Institute). Cells were cultured in RPMI 1640 medium supplemented with 1% l-glutamine 100 nM, 1% penicillin–streptomycin 100 U/ml and 5% fetal calf serum (Gibco) in a humidified incubator with 5% CO_2_. Most experiments were performed when cultures reached 80% confluence. To overexpress or knock-down Cofilin-1 in HK-2 cells, transfection was done with corresponding vectors using Lipofectamine 2000 (Invitrogen) following the manufacture’s protocol.

For Cofilin-1 overexpression, cofilin-1(NM_005507.3) coding sequences were amplified with the following primers and cloned into the pCDNA3.1 vector (Addgene). Forward, 5′-CCCAAGCTTATGGCCTCCGGTGTGGCTGTCTCTG -3′ (Hind III cutting site underscored); Reverse 5′-CGGAATTCTCACAAAGGCTTGCCCTCCAGGGAG-3′ (EcoR I cutting site underscored).

For siRNA constructs that target human Cofilin-1, the following primers were synthesized by Genepharma (Shanghai, China). siCFL1-1, 5′-GGGCAAGGAGAUUCUGGUATT-3′; siCFL1-2, 5′-GGAGAUUCUGGUAGGAGAUTT-3′; siCFL1-3, 5′-GCUCCAAGGAUGCCAUCAATT-3′; siNC (scramble siRNA), 5′-UUCUCCGAACGUGUCACGUTT-3′).

### Cell treatments

Cell injury was induced by OGD as previously described [21]. Briefly, cells were sequentially washed with warmed HEPES buffer and warmed OGD solution. Cells were then cultured in warmed OGD solution in an airtight chamber. After OGD treatment, cells were recovered for 24 h in a humidified 5% CO_2_ incubator at 37 °C after replacing the OGD solution with warmed culture medium.

### Cell proliferation assay

Cell proliferation assays were performed using Cell Counting Kit-8 (Dojindo Laboratories) following the manufacturer’s protocol. The absorbance at 450 nm (OD_450nm_) was measured by the Multiskan MS plate reader (Labsystems) to determine cell viability.

### RNA isolation and quantitative RT-PCR

The total RNA was extracted using Trizol reagent (Invitrogen) according to the manufacturer’s instructions. Quantitative RT-PCR was performed using SYBR®Green (Thermo Fisher Scientific) on the ABI 7300 instrument (Applied Biosystems). Target gene expression was normalized to the internal control (GAPDH) using the ^△△^CT formula. All data represented the mean of biological triplicates. The following primers were used: KIM-1, 5′ TTCCGTGTCTCTAAGATTG 3′ and 5′ TAATGGGTGTGACTCTATG 3′; NGAL, 5′ GGCAGGTGGTACGTTGTGG 3′ and 5′ GGTTGTAGTCCGTGGTGGC 3′; GAPDH, 5′ ATTCAACGGCACAGTCAAGG 3′ and 5′ CAGAAGGGGCGGAGATG 3′.

### Preparation of total lysates, cytosolic fraction and nuclear extracts, and western blot analysis

The total lysates were prepared with RIPA buffer containing protease inhibitors (Beyotime). The cytosolic fraction and nuclear extracts were prepared with NE-PER™ Nuclear and Cytoplasmic Extraction Reagents (Thermo Fisher Scientific) according to the manufacturer’s instructions. Proteins were separated by SDS–polyacrylamide gels and electro-transferred onto nitrocellulose membranes (Millipore, Billerica). Proteins of interest were immunoblotted using standard protocol with the antibodies listed below and chemiluminescence was detected using the ECL system (Millipore).

### Detection of lactate dehydrogenase (LDH), malondialdehyde (MDA) and Fe^2+^

Cell injury was assessed by measuring LDH release using an LDH assay kit (Nanjing Jiancheng Bioengineering Institute, A2020-2) according to the manufacturer’s instructions. The extent of lipid peroxidation was determined by measuring MDA using a thiobarbituric-acid-based commercial kit (Nanjing Jiancheng Bioengineering Institute) according to the manufacturer’s instructions. The iron content was detected by Perls staining assay with an iron assay kit (Abcam, ab83366) according to the manufacturer’s instructions.

### Lipid peroxidation assay using flow cytometry

Lipid reactive oxygen species (ROS) levels were determined by C11-BODIPY assay according to the manufacturer’s instructions (Thermo Fisher Scientific, D3861). Briefly, cells were first incubated in medium with 10 mM C11-BODIPY for 1 h and then resuspended in PBS with 1% BSA. Flow cytometry analysis (FACS CantoTM II, BD Biosciences) was employed to determine lipid ROS levels.

### Mouse acute kidney injury (AKI) model

The care and use of laboratory animals in this work were in accordance with the National Institutes of Health Guide and were approved by the Research Ethics Committee at Shanghai Jiao Tong University First People’s Hospital (Permit number: 2013KY001). 6–8 weeks old male C57 BL/6 mice (20–22 g) were used in the experiments and housed under temperature- and humidity-controlled condition, with a 12 h/12 h light/dark cycle. Mice were anesthetized by intraperitoneally administering ketamine (100 mg/kg) and xylazine (10 mg/kg). Renal pedicles were bilaterally clamped for 25 min using nontraumatic microaneurysm clamps. Mice were kept on a heating pad to maintain constant body temperature at 37 °C and received analgesia after surgery. The control group was only given anesthesia and laparotomy. 50 μg of siRNA against mouse Cofilin-1 (siCLF1, 5′-GUGUCAUCAAGGUGUUCAATT-3′) or control siRNA (siNC, 5′-UUCUCCGAACGUGUCACGUTT-3′) was administered through the caudal vein immediately after reperfusion (*n* = 6 for each group). After 48 h, kidneys and blood samples were harvested in each group.

### Renal function assay

Serum creatinine and blood urea nitrogen (BUN) levels were measured using commercially available kits (Nanjing Jiancheng Bioengineering Institute).

### Histology and immunohistochemistry

Tissues of interest (left kidney) fixed in 10% formalin were dehydrated using graded ethanol series before they were embedded in paraffin. The tissue blocks were then cut into 4 μm sections and subjected to histopa-thological examinations using hematoxylin and eosin (H&E) and periodic acid Schiff (PAS) staining techniques. Immunohistochemistry analysis was performed as previously described [22] using antibodies listed below.

### Antibodies and reagents

For western blotting, anti-Cofilin-1 (Abcam, ab124979), anti-p-Cofilin-1(Ser3) (Abcam, ab12866), anti-ATF4 (Abcam, ab23760), anti-CHOP (Cell Signaling Technology, #2895), anti-DRP1 (Cell Signaling Technology, #8570), anti- NF-κB p65 (Cell Signaling Technology, #8242), anti-H3 (Cell Signaling Technology, #8173), anti-GAPDH (Cell Signaling Technology, #5174) and HRP-conjugated rabbit secondary antibodies (Beyotime, Shanghai, China).

The NF-κB inhibitor pyrrolidine dithiocarbamate (PDTC) was purchased from MCE. ER stress inhibitor GSK2606414 and ferroptosis inhibitor Fer-1 were purchased from Selleck.

### Statistical analysis

Statistical analysis was carried out using Graphpad Prism 6.0. ANOVA tests were carried out and p < 0.05 were considered significant.

## Results

### *Establishing an AKI model *in vitro

**Fig. 1:**
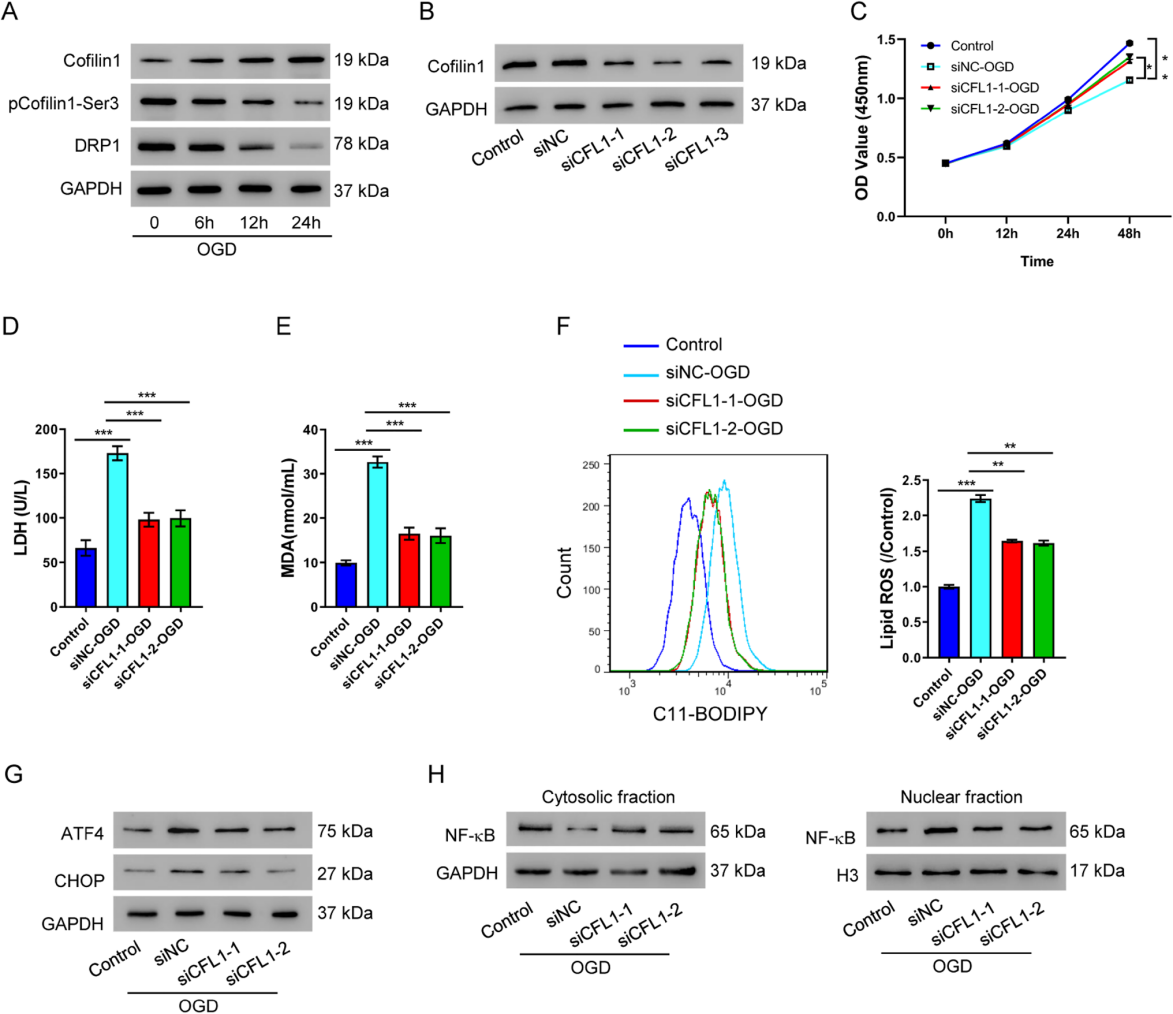
Cofilin knock-down alleviates OGD-induced injuries in cultured HK-2 cells. **A** Western blot of Cofilin-1, phosphorylated Cofilin-1 (Ser3) and DRP1 after oxygen-glucose deprivation (OGD) treatment for indicated time periods. **B** Western blots show efficient knock-down of Cofilin-1 after cells were transfected with three siRNAs (siCLF1-1, siCLF1-2, siCLF1-3, respectively), when compared with untransfected cell (control) and cells transfected with scramble siRNA (siNC). In panels (**C**–**H**), HK-2 cells were first transfected with Cofilin-1 siRNAs (siCLF1-1 or siCLF1-2) before OGD treatment. **C** Cell viability assay at indicated time points. **D** LDH release assay 48 h after OGD treatment. **E** MDA content. **F** Lipid ROS. **G** Western blot of ATF4 and CHOP. **H** Western blot of cytosolic and nuclear NF-κB p65. *P < 0.05, **P < 0.01, ***P < 0.001

We first determined the level of Cofilin-1 expression in OGD-treated cells. We found that oxygen-glucose deprivation upregulated total Cofilin-1 in a time-dependent manner (Fig. 1A). Significantly, the inactive form of Cofilin-1, phosphorylated Cofilin-1, was downregulated after OGD treatment (Fig. 1A), suggesting OGD treatment could upregulate active Cofilin-1 levels, presumably its activity as well.

### Silencing Cofilin-1 alleviates OGD-induced cell injury

**Fig. 2:**
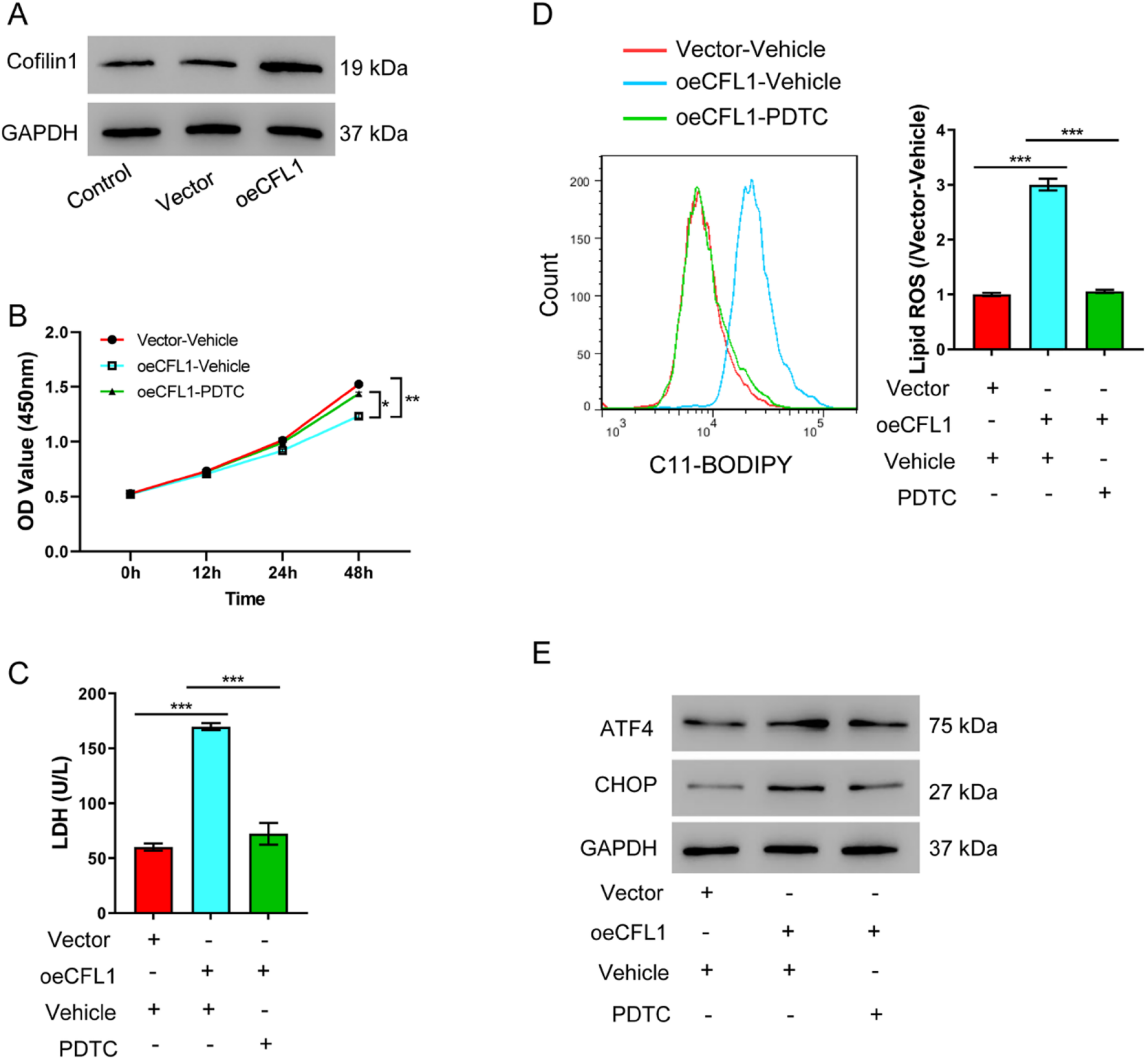
Cofilin-1 induces AKI via the NF-κB pathway. **A** Western blot showing successful overexpression of Cofilin-1 with the oeCFL1 construct. **B**–**E** Cofilin-1 expressing HK-2 cells were exposed to 10 µM NF-κB inhibitor PDTC before the following assays were performed: **B** Cell viability assay at different time points. **C** LDH release assay 48 h after transfection. **D** Lipid ROS levels 48 h after transfection. **E** Western blot of ATF4 and CHOP 48 h after transfection. *P < 0.05, **P < 0.01, ***P < 0.001

We designed three independent siRNAs to perform loss-of-function analysis of Cofilin-1. When compared with the control group, all three siRNAs efficiently knocked down Cofilin-1 protein levels. siCFL1-1 and siCFL1-2 were used for follow-up experiments, as they exhibited higher knock-down efficacy (Fig. 1B). To determine the potential role of Cofilin-1, we examined the effect of Cofilin-1 knock-down on OGD treatment. Interestingly, knocking down Cofilin-1 had protective effect in OGD-induced AKI cells. Although cells had significantly diminished viability after OGD treatment for 48 h, knocking down Cofilin-1 before OGD treatment protected cells from such viability loss (Fig. 1C). In addition, we also assessed cell injury by quantifying LDH release and found OGD treatment increased cell injury, which was also alleviated by silencing Cofilin-1 (Fig. 1D). These results suggested Cofilin-1 was likely to mediate the injury signals induced by OGD treatment, and that silencing Cofilin-1 could protect cells from OGD treatment. Ferroptosis is an oxidative cell death associated with increased lipid peroxidation. OGD-induced cell injury increased lipid ROS, whereas knocking down Cofilin-1 alleviated increased ROS (Fig. 1E, F). OGD treatment also increased ER stress in HK-2 cells, as indicated by increased ATF4 and CHOP protein levels. Silencing of Cofilin-1 in HK-2 cells ameliorated ER stress in AKI (Fig. 1G). We finally determined the cytosolic and nuclear fractions of NF-κB upon Cofilin-1 manipulation. A significant portion of NF-κB was translocated to the nucleus upon OGD-induced AKI (Fig. 2H). Knocking down Cofilin-1 reversed the nuclear accumulation of NF-κB, as compared to the results in the control group. Taken together, we concluded silencing Cofilin-1 alleviated OGD-induced cell injury phenotypes.

### Inhibiting NF-κB signaling reduces Cofilin-1-induced cell injury

To further investigate the possible mechanism by which Cofilin-1 mediated AKI, we examined the effects of Cofilin-1 overexpression (Fig. 2A). We first confirmed that Cofilin-1 overexpression induced hallmarks of cell injury, including decreased cell viability and LDH release, increased lipid peroxidation and ER stress. To explore if Cofilin-1 was inducing cell injury via NF-κB signaling, we examined the effect of PDTC which is well-established to inhibit NF-κB [22, 23]. We found that Cofilin-1-overexpressing cells which are exposed to PDTC had normal cell viability without obvious cell injury phenotypes (Fig. 2B, C), and normal levels of lipid ROS and ER stress which are comparable to cells without PDTC (Fig. 2D, E). Blocking NF-κB rescued the effects of Cofilin-1 overexpression. These results suggested inhibiting NF-κB signaling reduces Cofilin-1-induced cell injury.

### Reliving ER stress reduces Cofilin-1-induced cell injury

**Fig. 3:**
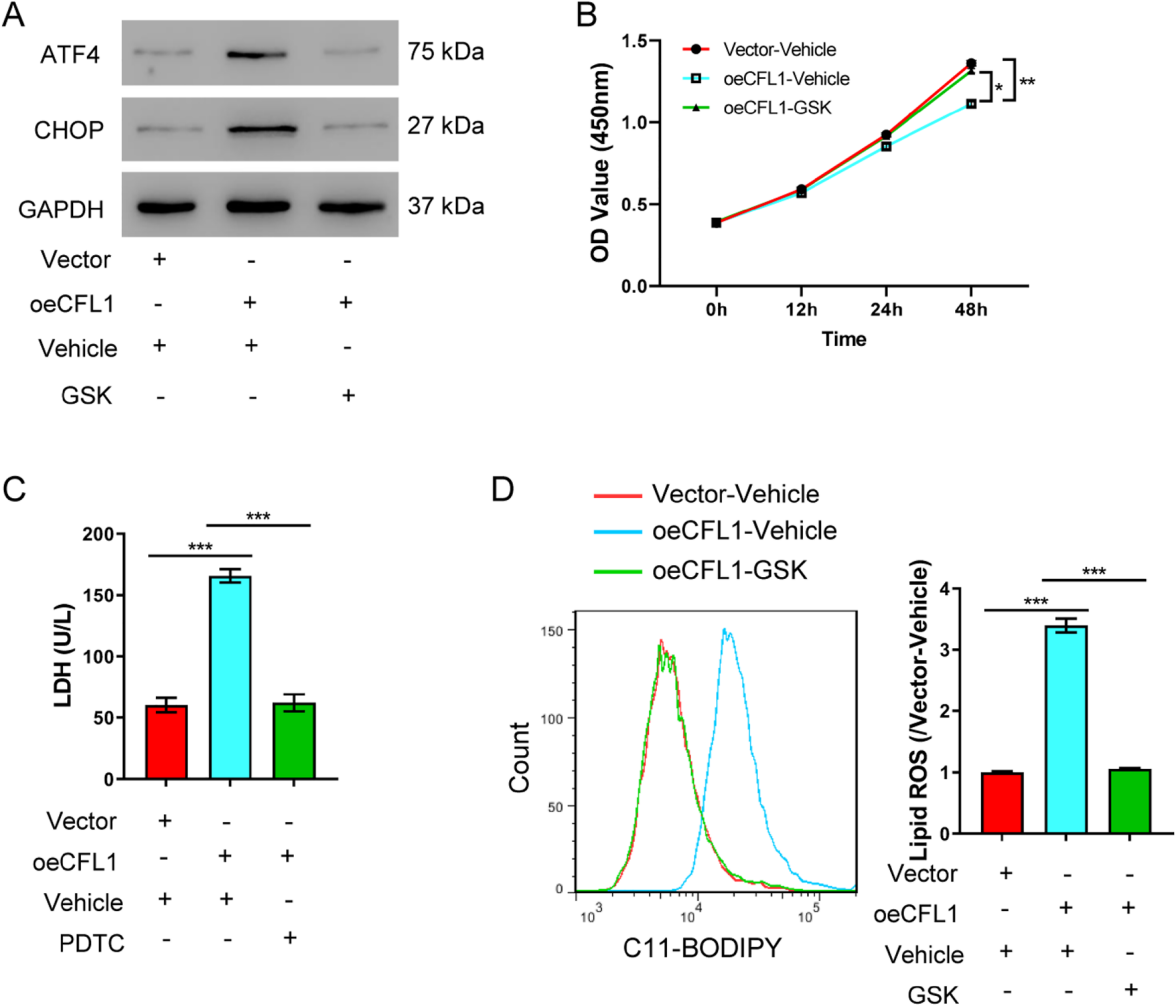
Cofilin-1 induces AKI via the ER stress pathway. HK-2 cells were transfected with the Cofilin-1 overexpression construct before exposed to 1 µM ER stress inhibitor GSK2606414. **A** Western blot of ATF4 and CHOP 48 h after transfection. **B** Cell viability assay at different time points. **C** LDH release assay 48 h after transfection. **D** Lipid ROS levels 48 h after transfection. *P < 0.05, **P < 0.01, ***P < 0.001

As our AKI model also featured increased ER stress, we next explored if Cofilin-1 induced cell injury was regulated through ER stress. GSK2606414, a protein kinase RNA-like ER-kinase inhibitor, is known to extinguish ER stress [24], was used to testify it (Fig. 3A). Similar with the results above, Cofilin-1 overexpression decreased cell viability (Fig. 3B) while increased LDH release (Fig. 3C) and lipid ROS levels (Fig. 3D). Remarkably, the ER stress inhibitor rescued Cofilin-1 overexpression by restoring cell viability and lipid peroxidation to normal levels. The results suggested reliving ER stress could attenuate Cofilin-1-induced cell injury.

### Inhibiting ferroptosis ameliorates Cofilin-1-induced cell injury

**Fig. 4:**
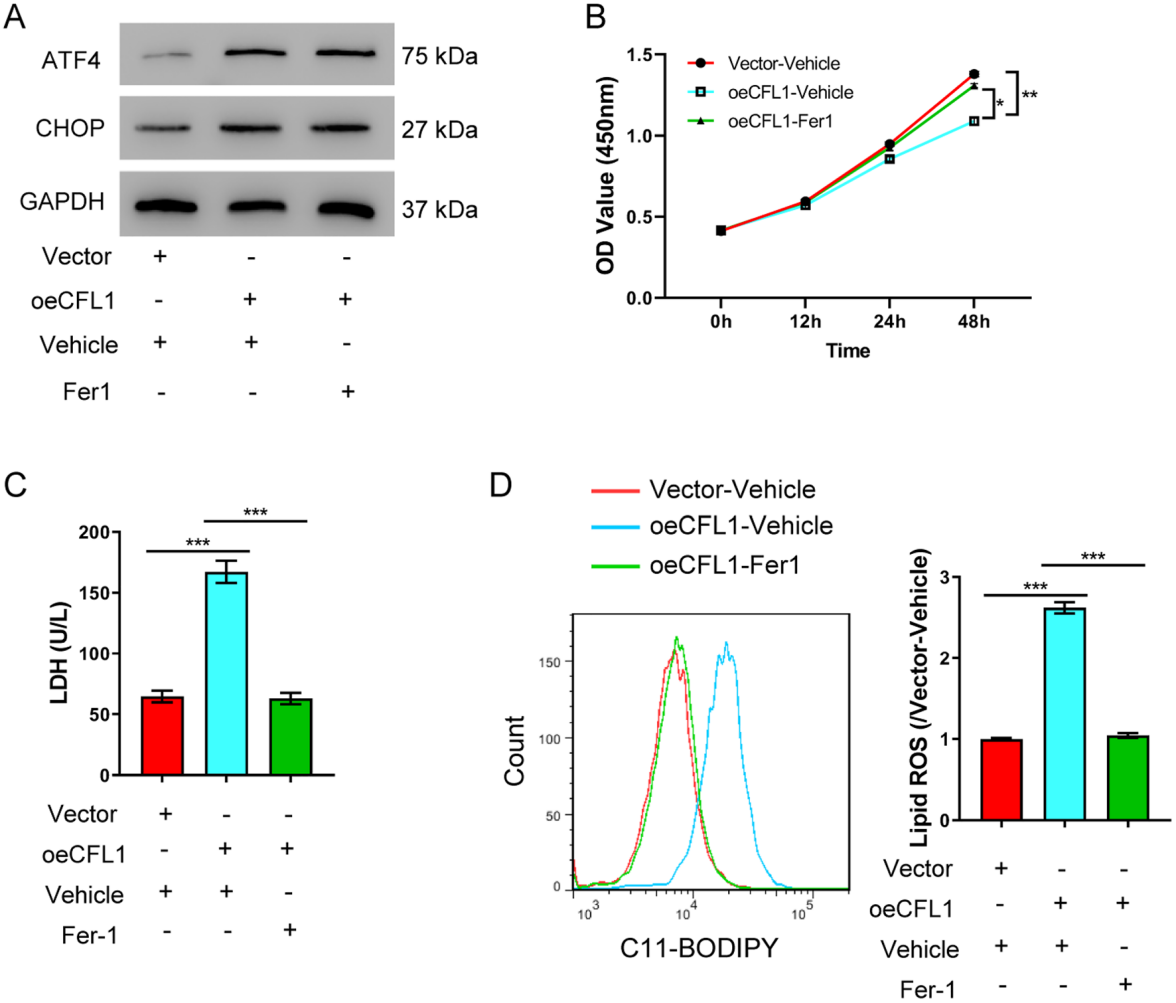
Cofilin-1 induces AKI by promoting ferroptosis. HK-2 cells were transfected with the Cofilin-1 overexpression construct before exposed to 2 µM ferroptosis inhibitor Fer-1. **A** Western blot of ATF4 and CHOP 48 h after transfection. **B** Cell viability assay at different time points. **C** LDH release assay 48 h after transfection. **D** Lipid ROS levels 48 h after transfection. *P < 0.05, **P < 0.01, ***P < 0.001

As discussed above, ferroptosis has been implicated in a wide range of disease contexts [7]. We therefore investigated if ferroptosis was contributed to Cofilin-1-induced cell injury. To directly assess ferroptosis in Cofilin-1-induced cell injury, we took advantage of a specific ferroptosis inhibitor, ferrostatin-1 (Fer-1). We found that Fer-1 treatment reverted Cofilin-1-induced cell injury. Treating Cofilin-1-overexpressing cells with Fer-1 rendered cell to have normal viability (Fig. 4B, C). We also found that Fer-1 reduced lipid peroxidation to normal levels, which was otherwise increased by Cofilin-1 overexpression alone (Fig. 4D), suggesting that inhibiting ferroptosis reduced Cofilin-1-related cell injury. Interestingly, Fer-1 had indiscernible effect on Cofilin-1-induced ER stress, as indicated by unchanged ATF4 and CHOP levels (Fig. 4A). These results together argued that ferroptosis was indeed contributed to Cofilin-1-induced lipid peroxidation and cell injury, probably downstream of or in parallel to the ER stress pathway.

### Cofilin-1 knock-down mitigates IRI-induced AKI in vivo

**Fig. 5:**
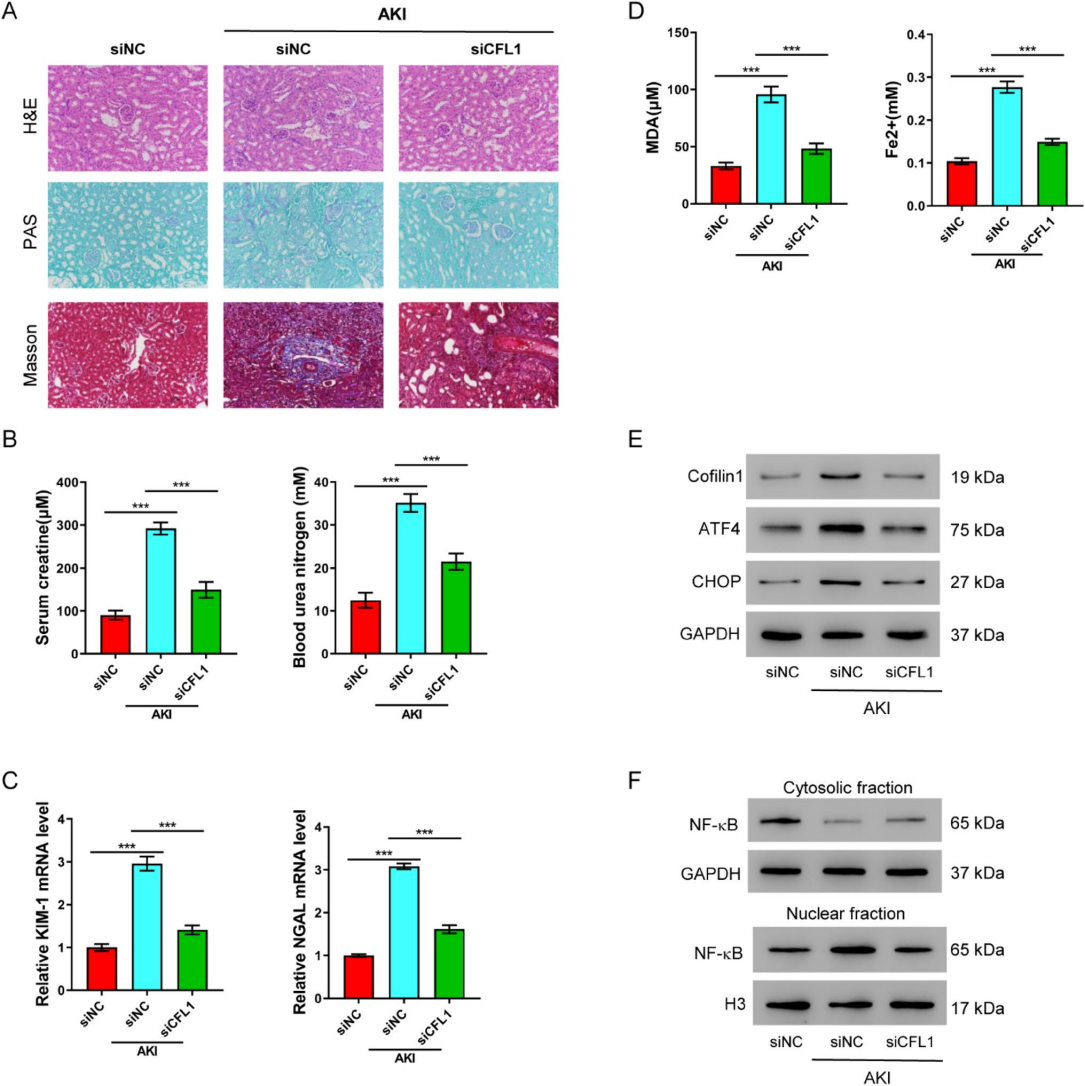
Cofilin-1 knock-down ameliorates IRI-induced AKI in vivo. Mouse model of IRI-induced AKI was established and subject to Cofilin-1 siRNA (siCFL1) treatment. **A** Hematoxylin and Eosin (H&E) and Periodic Acid Schiff (PAS) staining, 200 × magnification. **B** Serum creatinine and blood urea nitrogen levels. **C** Expression of renal tubular damage markers, KIM-1 and NGAL quantified by qRT-PCR. **D** MDA and Fe^2+^. **E** Western blot of Cofilin-1, ATF4 and CHOP in renal tissues. **F** Western blot of cytosolic and nuclear NF-κB p65 in renal tissues. ***P < 0.001

To validate our above findings in vitro, we established an IRI-induced AKI model in mice. Histological examination revealed severe renal damages upon tissue injury, which were characterized by abnormal tubular morphology and significant luminal debris. Promisingly, when AKI mice administered with siRNA to silence Cofilin-1 immediately after reperfusion, their kidney tissues were significantly improved (Fig. 5A). Similar results were born out by various renal function assays. Although mice with AKI had markedly higher serum creatinine and blood urea nitrogen, silencing Cofilin-1 functionally rescued their kidneys by lowering both serum creatinine and blood urea nitrogen (Fig. 5B). Consistently, inhibiting Cofilin-1 in vivo also alleviated renal damages, as shown by lower expression of kidney damage markers (Fig. 5C), lower MDA and Fe^2+^ levels (Fig. 5D). Altogether, Cofilin-1 knock-down mitigated IRI-induced AKI and improved renal function in vivo*,* possibly by regulating ER stress and NF-κB signaling pathways.

## Discussion

Due to its ever-increasing prevalence, AKI has become a main factor of hospitalized patients morbidity and mortality [2]. However, we are still lacking for a complete understanding of AKI pathogenesis and faithful markers for early diagnosis. The most important finding of the current study is the identification of possible mechanism by which Cofilin-1, a newly proposed biomarker for AKI, induces AKI and its link to previously established pathways that are involved in AKI initiation and progression.

Cofilin-1 belongs to a protein family that are known to regulate cytoskeletal dynamics [18]. It has been shown that Cofilin-1 is an important renal epithelial architecture maintenance and therefore critical for recovery from kidney injury [25]. Moreover, urinary Cofilin-1 is significantly upregulated in AKI patients, and in cultured kidney injury models [18, 20]. We initiated this study by examining the expression of Cofilin-1 in our OGD-induced AKI models and found OGD treatment upregulated the levels of total Cofilin-1 and, perhaps more importantly, of its active, unphosphorylated form. Significantly, inhibiting Cofilin-1 by siRNAs could substantially rescued all the AKI-related phenotypes we examined. Knocking down Cofilin-1 could not only restore the cell viability loss resulted from OGD-induced AKI, but also reduce ferroptosis, ER stress and nuclear NF-κB translocation. All the current studies, including ours, provide support to the notion that high Cofilin-1 levels are associated with AKI. Nevertheless, little is known about how Cofilin-1 overexpression could contribute to AKI.

Cofilin-1 has been reported to regulate NF-κB signaling in various contexts. For example, Cofilin-1 could act downstream of the RhoA/Rho-associated kinase (ROCK) pathway to regulate actin filaments dynamics and activate NF-κB signaling in endothelial cells. Knocking down Cofilin-1 could stabilize actin and diminish NF-κB activity that is otherwise activated by RhoA [26]. Studies have also shown that silencing Cofilin-1 could reduce the nuclear fraction of NF-κB/p65 in AngII-treated kidney cells [27]. Moreover, Cofilin-1 is also essential for NF-κB and JAK-STAT activities in LPS-induced microglial cell activation [28]. We therefore examined if this regulatory axis sustained in AKI using an NF-κB inhibitor. We demonstrated that NF-κB inhibitor could abolish the effects of Cofilin-1 overexpression, suggesting Cofilin-1 is inducing AKI and other cellular defects via the NF-κB pathway.

In addition, Impeded ER homeostasis and increased ER stress were also evident in our AKI model, as indicated by increased levels of activating transcription factor 4 (ATF4) and C/EBP homologous protein (CHOP). We further discovered that Cofilin-1 overexpression led to elevated ER stress and that inhibiting ER stress pathways also voided the effect of Cofilin-1 overexpression. In fact, emerging evidence has shown interactions between unfolded protein response (UPR) and NF-κB at transcriptional, post-transcriptional and post-translational levels [29]. Indeed, some studies have shown that ER stress were under the control of the NF-κB pathway [27]. It would of great interest to dissect how these two pathways communicate in the context of AKI.

Ferroptosis, an iron-dependent, non-apoptotic cell death program, has drawn a wide range of attention since its first discovery in 2012 [4], and found that involved in the development of AKI [8]. Studies have revealed that ER stress might be a trigger for ferroptosis in cadmium-exposured renal tubular cells [28]. To further explore the role of ferroptosis in AKI, we employed a chemical inhibitor approach. We found that inhibiting ferroptosis could override Cofilin-1 overexpression. More interesting, the ferroptosis inhibitor modulate Cofilin-1 induced AKI in an ER-stress-independent manner, suggesting ferroptosis is either downstream of or in parallel to ER stress signaling in the context of AKI. The former notion of crosstalk between ER stress and ferroptosis is plausible and several studies have provided evidence. For example, ferroptosis could induce ER stress by activating UPR, which in turn activates the PERK-ATF4/CHOP axis to initiate the transcription of downstream targets, including those participating in cell apoptosis [30–32]. In a different example, researchers demonstrated that genetic and pharmacological inhibition intracellular cystine-glutamate antiporter led to both ER stress and ferroptosis, although the detailed mechanism merit further studies [33]. On the other hand, erastin could trigger ER stress-independent ferroptosis in melanoma cells [34], it would be interesting to determine the dependence of ferroptosis on ER stress in this context.

## Conclusion

In conclusion, this study has revealed key functional mechanisms of Cofilin-1 in inducing ferroptosis in AKI, probably via regulating NF-κB and ER stress in our in vitro and in vivo models. Further validation will be done in human patient to provide clinical relevance and insights in new therapeutic strategies for the treatment of AKI.

### Supplementary Information

Below is the link to the electronic supplementary material.

Supplementary file1 (DOCX 1360 KB)

## Data Availability

The data that support the findings of this study are available on request from the corresponding author, Yingjian Zhu, upon reasonable request.
