# Supplementary figures and images for: Cofilin-1 induces acute kidney injury via the promotion of endoplasmic reticulum stress-mediated ferroptosis
Source: Hum Cell. 2023 Aug 7;36(6):1928–37. doi: 10.1007/s13577-023-00949-9 (PMC10587211; doi:10.1007/s13577-023-00949-9)

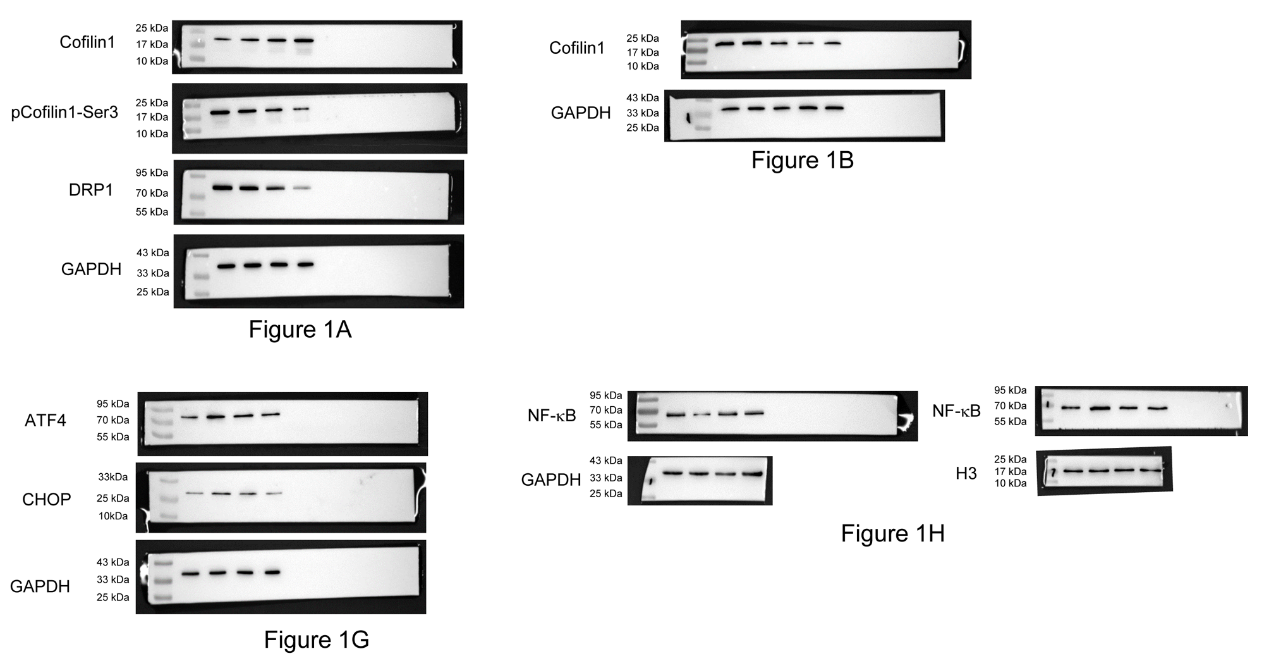


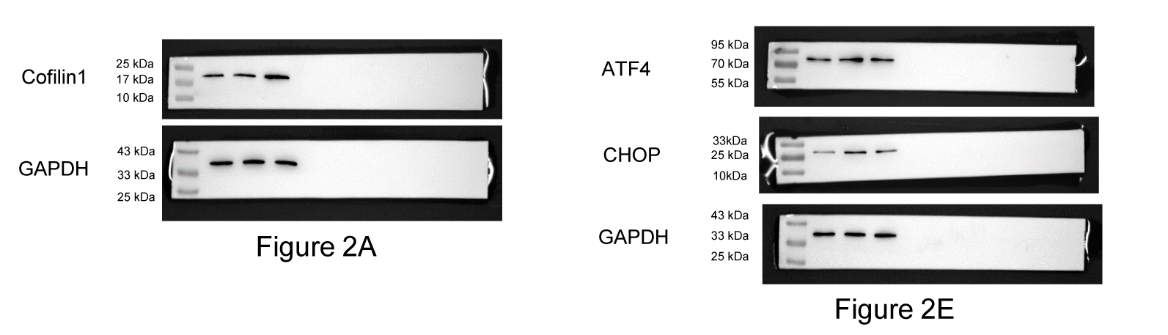


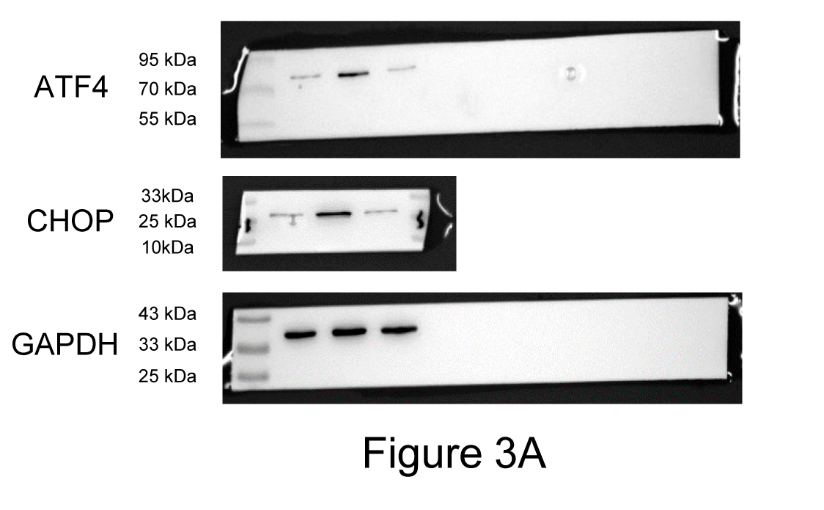


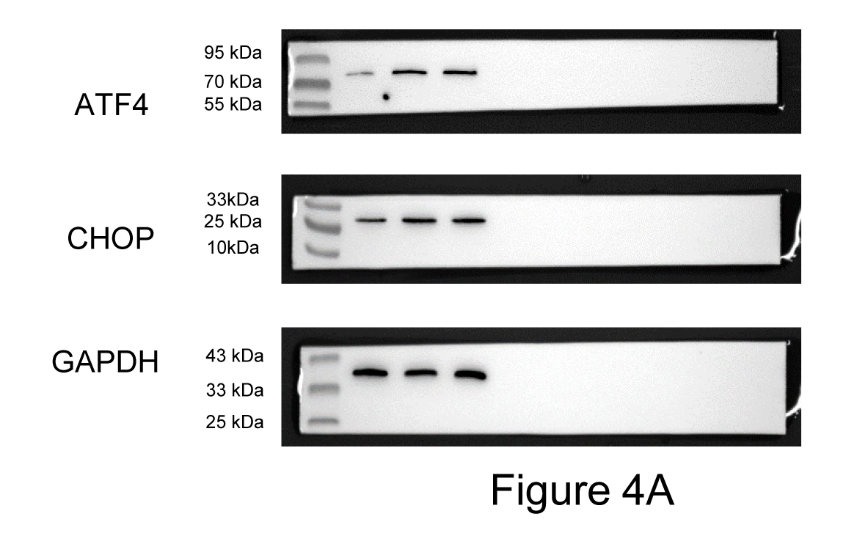


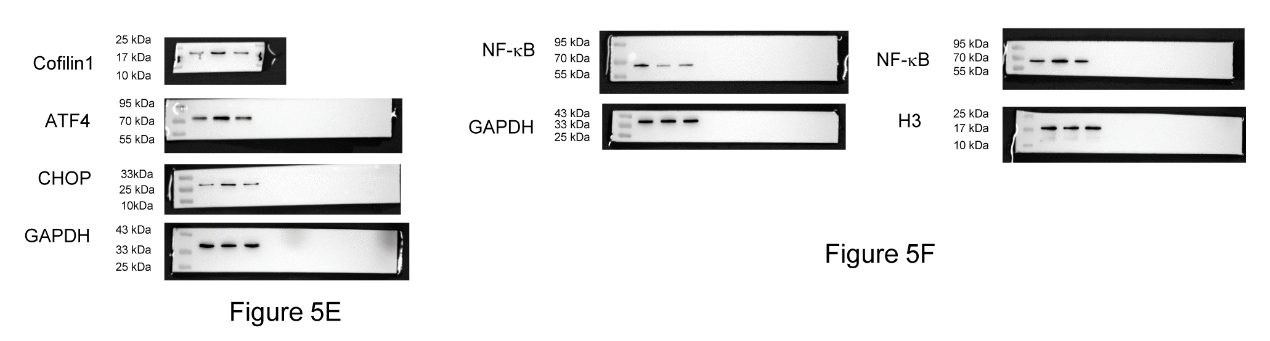

Supplement: Supplementary file 1 — Supplementary file1 (DOCX 1360 KB) [file 13577_2023_949_MOESM1_ESM.docx]
